# With phases: how two wrongs can sometimes make a right

**DOI:** 10.1107/S0907444909048112

**Published:** 2010-03-24

**Authors:** Pietro Roversi, Steven Johnson, Susan M. Lea

**Affiliations:** a Sir William Dunn School of Pathology, University of Oxford, South Parks Road, Oxford OX1 3RE, England

**Keywords:** molecular replacement, experimental phasing, phase combination

## Abstract

In isolation, both weak isomorphous/anomalous difference signals from heavy-atom derivatization and phases from partial molecular-replacement solutions for a subset of the asymmetric unit often fall short of producing interpretable electron-density maps. Phases generated from very partial molecular-replacement models (if generated carefully) can be used to reliably locate heavy-atom sites, even if the signal is not sufficiently strong to allow robust finding of the sites using Patterson interpretation or direct methods. Additional advantages are that using molecular-replacement phases to define the heavy-atom substructure avoids the need for subsequent hand determination and/or origin-choice reconciliation and that the partial model can be used to aid the mask determination during solvent flattening. Two case studies are presented in which it was only by combining experimental and molecular-replacement phasing approaches that the crystal structures could be determined.

## Abstract 2

Combining experimental phases and those from refinement of very incomplete models significantly improves electron-density maps.

## 1. General principles and computer programs used

When we learn how to determine crystal structures, we traditionally think of there being two major routes to phase determination: ‘experimental’ or ‘molecular replacement’. However, in a minority of cases we can get stuck when, whatever is performed in data collection and analysis, the quality of the maps obtained from either of these processes is not sufficient to generate an ‘interpretable’ map. Here, we present two case studies in which it was only by combining both phasing approaches (and a certain degree of intransigence) that the structures were determined. Combining experimental phases in refinement is by no means a new concept: for a review of the advantages that phase combination brings to macromolecular refinement, see Pannu *et al.* (1998 ▶), Adams *et al.* (2009 ▶) and references therein. This article does not set out to be a tutorial in either experimental phasing or molecular-replacement theories or practical methodologies; there are many good articles that already fill this ecological niche (*e.g.* Dodson, 2003 ▶, 2008 ▶; Dauter *et al.*, 2002 ▶; Perrakis *et al.*, 2001 ▶). Rather, we hope that this small contribution will fit in the by now time-honoured tradition of case-study papers in issues of *Acta Crystallographica Section D* devoted to the proceedings of the CCP4 Study Weekend (Sanders *et al.*, 2001 ▶; Stewart *et al.*, 2008 ▶; Esnouf *et al.*, 2006 ▶; Cowan-Jacob *et al.*, 2007 ▶; Jenni & Ban, 2009 ▶; Rudenko *et al.*, 2003 ▶; Calderone, 2004 ▶). We also do not wish to seek to claim that the methods we present are the only ones by which these structures could have been determined, but they are the way that we determined them. Nor have we performed a close comparison of whether different computer programs could have been used to accomplish the various computational steps: these are real case studies and we present the results obtained from the software we used and our rationale for choosing that software, bearing in mind that at least part of any rationale for software choice is always familiarity. The flexibility of the *SHARP* (Vonrhein *et al.*, 2007 ▶) experimental phasing suite and in particular the ease with which an external source of phases can be input to allow heavy-atom finding on a common origin to another set of phases, together with incorporation of these ‘external’ phases in the refinement of the heavy-atom model, led us to use *SHARP* for our experimental phase determination. Similarly, it has previously been demonstrated that where molecular-replacement models are highly partial (*i.e.* less than 60% complete), modelling the ‘missing atoms’ as part of refinement significantly improves the quality of the maps obtained (Blanc *et al.*, 2004 ▶). In this way, *BUSTER-TNT* has the advantage that its low-resolution *F*_calc_ can include a con­tribution from the part of the structure that is ordered but is yet to be modelled (the ‘missing atoms’); this in turn makes the scaling more accurate and decreases the model bias that is normally introduced by the refinement of partial structure parameters against data that contain scattering from the entirety of the asymmetric unit. In our hands, even at the earliest stages of refinement with models constituting only one third of the structure the use of missing-atom modelling in *BUSTER-TNT* was viable if phase-combined maps were used to define the missing atoms’ prior distribution. The latter was accomplished by using a combined *BUSTER-TNT*/*SHARP* solvent-flattened map (the phases were combined in *SHARP* and solvent flattening was carried out with *SOLOMON* launched from within the *SUSHI* interface) to define the missing-atoms envelope during refinement.

## 2. Case studies

Both case studies presented here involve the determination of structures that include domains of the human complement regulator factor H (fH). fH is a classic ‘beads-on-a-string’ molecule consisting of 20 sequential short consensus repeat domains (SCRs) connected by linker sequences of between one and five residues. fH is an important component of the innate immune system that acts to regulate the destructive power of the complement system and prevent harm to our own cells. fH acts both by dissociation of a cell-surface molecule (C3bBb; the so-called ‘decay-accelerating’ activity) and also by assisting the direct cleavage of the same molecule by another enzyme, complement factor I (the so-called ‘co­factor’ activity). These activities are carried out *via* a variety of protein–protein and protein–carbohydrate inter­actions that occur at different sites along the extended fH molecule. Prior to the work on the fH constructs reviewed here, the structures of many SCR domains were known (both from fH and from other proteins) and some information was available about the overall fH architecture from both electron microscopy and small-angle X-ray scattering, but there was no atomic structural information about the sixth to eighth SCR domains, which are involved in various interesting biological roles. SCR domains are about 60 residues long and contain two conserved disulfide bridges and a conserved tryptophan. There are about 30 independent atomic structures of SCRs available in the Protein Data Bank (Soares *et al.*, 2005 ▶). Therefore, when initiating the factor H crystallographic projects described here, at least two obvious modes of obtaining phasing information presented themselves: firstly, molecular replacement would seem an entirely reasonable approach and, secondly, in the event of failure of that approach sulfur-SAD could potentially be used.

### 2.1. Case study 1: the structure of SCRs 6, 7 and 8 from the complement regulator factor H

Although the structures of several fH SCR domains were already known, we were interested in the structure and arrangement of domains in the region of SCR domain 7, since this region is implicated in recognition of surface glycosaminoglycans, thus conferring on fH the selectivity that enables it to protect self-tissues against complement-mediated lysis. In order to obtain structural insight into the fH–glycosamino­glycan recognition event, crystals were grown of a three SCR-domain construct comprising domains 6, 7 and 8 (fH678) in complex with sucrose octasulfate (SOS; Prosser, Johnson, Roversi, Herbert *et al.*, 2007 ▶), a highly sulfated analogue of glycosaminoglycans.

Crystals of fH678–SOS were obtained and native data were collected to 2.4 Å resolution; the crystals belonged to space group *C*222_1_ and had a likely content of one copy of fH per asymmetric unit (Prosser, Johnson, Roversi, Clark *et al.*, 2007 ▶). We then sought to use all possible SCR domains from the PDB to determine the structure by three different molecular-replacement protocols: three sequential searches for single SCR search models, a search for a pair of SCR domains followed by an additional single one or a single search for a triple SCR-domain search model. The molecular-replacement computer programs used were *MOLREP*, *Phaser* and *AMoRe* from *CCP*4. Various modifications of the models, including the use of *CHAINSAW* (Stein, 2008 ▶), reducing to the backbone and forming ensembles, were tried. None of these attempts proved successful. This was rationalized as arising from the fact that whilst it was clear that the three domains we were searching for were SCR domains (each with a pair of conserved disulfides and a buried tryptophan), the level of sequence identity was low (20–30%). When different SCR models are overlaid, it is clear that these domains pack a lot of structural variation into a small and fairly constrained structure. SCR interdomain angles also vary widely and cannot be predicted. These orthorhombic crystals suffered rapid radiation damage, thus hindering attempts at phasing by sulfur-SAD (which needs high-redundancy data sets, preferably collected from a single crystal) or phasing by radiation damage-induced phasing (RIP; Ravelli *et al.*, 2003 ▶) (which needs a relatively radiation-damage-free data set before the non-isomorphism is introduced by a heavy X-ray dose and still requires measurable diffraction from the same crystal afterwards). As FH_678_ con­tains two methionines (one in SCR domain 6 and one in SCR domain 7), SeMet labelling of the recombinant protein was performed. Multiple data sets were collected (for details of data quality, see Prosser, Johnson, Roversi, Clark *et al.*, 2007 ▶) and *autoSHARP*–*SHELXD* could interpret both the anomalous and the isomorphous difference Patterson maps with a model containing two Se atoms. *SHARP* was used to refine the heavy-atom model against a 2.4 Å resolution native data set and a three-wavelength 2.5 Å resolution SeMet MAD data set and *SOLOMON* was used to solvent-flatten the resultant phases, but the correct hand could not be distinguished. The Patterson peak heights and the heavy-atom parameters refined for these two sites in *SHARP* suggested that one of the methionines was significantly more ordered than the other and even the more ordered methionine was undergoing a large thermal motion: the *SHARP* *B* factor for one Se refined to around 100 Å^2^ and the *B* factor for the other Se refined to around 170 Å^2^. These *B*-factor values were obtained consistently against isomorphous and anomalous differences from a number of different SeMet MAD data sets and irrespective of the treatment of heavy-atom site occupancies during the refinement protocol; both letting individual Se occupancies vary while keeping *f*′ and *f*′′ at the experimentally measured values and keeping the occupancies fixed to unity while refining *f*′ and *f*′′ instead produced the same result. The final refined *B* factors for the S atoms of these Met residues in the deposited crystal structure are 52 and 90 Å^2^ (the average *B* factor for the side chain was 50 Å^2^). The lack of hand discrimination power of this set of experimental phases was therefore not unexpected, since attempting to phase a 186-residue structure with essentially one poorly ordered Se atom was rather beyond the realms of the possible. Attempts to use heavy-atom soaks to generate additional phasing information also failed.

At this point new phase information was needed and it was provided in the form of the structure of SCR 7 (hereafter known as fH7), which was determined by Paul Barlow and colleagues using NMR (Herbert *et al.*, 2007 ▶). A single copy of a search model from the NMR ensemble could unambiguously be found using each of the molecular-replacement programs: the MR hit (as became obvious with hindsight after structure completion) was always to the fH7-domain placement and repeating the search with the same fH7 model (or any of the other SCR domains from the PDB in the presence of the fH7 first partial model) did not reveal the position and orientations of the flanking SCR domains. Refinement of the fH7 model (consisting of less than a third of the structure) against the fH678–SOS data using *BUSTER-TNT* led to maps which were not readily interpretable outside of the modelled domain.

**Figure 1:**
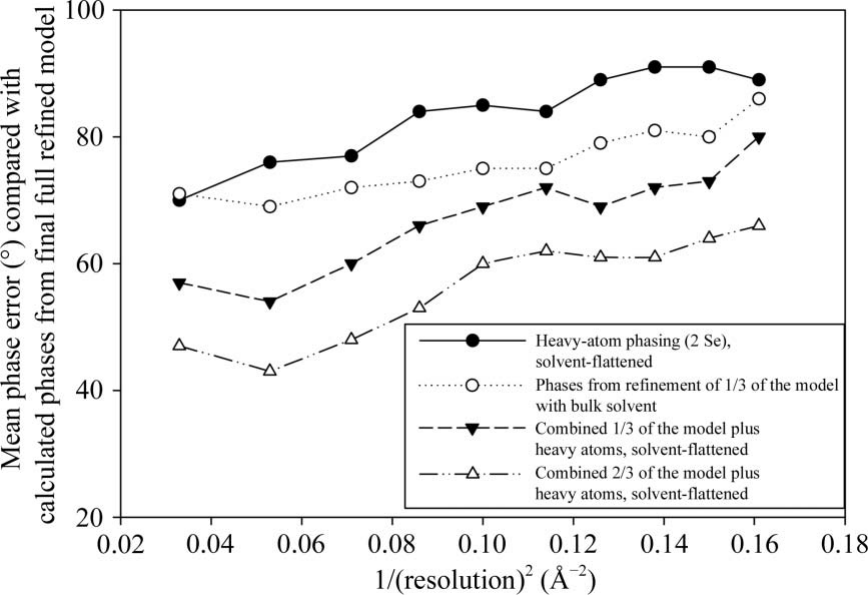
Mean phase error across the resolution range for fH678 phase sets at various stages of structure solution compared with calculated phases from the final complete model.

**Figure 2:**
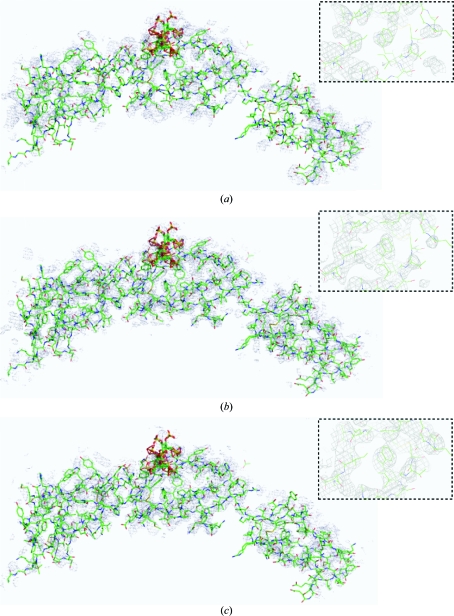
Electron-density maps calculated at various stages of the fH678 structure solution with the final coordinates displayed as stick models. The inset shows a close-up of a typical portion of the map in a region for which no molecular model existed at the point of calculating the map. (*a*) A *SOLOMON* solvent-flattened map derived from *SHARP* phase distributions generated from SeMet anomalous data. (*b*) *BUSTER-TNT* phases generated by refining fH7 against the fH678 data with missing atoms modelled. (*c*) *SOLOMON* solvent-flattened map using *SHARP* phase distributions generated from SeMet anomalous data in which *BUSTER-TNT* phase distributions (as in *b*) were used within the *SHARP* refinement and for calculation of *SHARP* output phase distributions.

At this point we were therefore in possession of two sets of poor phases and so it became an obvious strategy to attempt to combine these phase distributions to generate improved phase estimates (Read, 1986 ▶). Various methods for combining the experimental and molecular-replacement phases were con­sidered, but for ease of resolution of hand/origin issues and also to help the conditioning of the heavy-atom refinement we decided to use the *DM* solvent-flattened *BUSTER-TNT* phases as input to *SHARP* and to use them for heavy-atom location, heavy-atom refinement and to generate the final phase distributions. These phases were good enough to con­firm the location of both Se atoms in the isomorphous and anomalous log-likelihood gradient (LLG) maps in *SHARP*; more importantly, refinement of the two-Se heavy-atom model conditioned by the *BUSTER-TNT* phases and solvent flattening of the internally combined phase distributions (using *SOLOMON* as driven from the *SUSHI* interface) produced a new ‘phase-combined and solvent-flattened’ map that began to show sensible electron density outside the fH7 model and allowed the building of some residues at the N- and C-termini of the central domain. It is worth noting the phase combination with the model phases prior to solvent flattening had the additional advantage that the solvent-flattening masks included the parts of structure modelled thus far. Fig. 1 ▶ shows the phase errors between both the uncombined and combined phases compared with the reference phases generated from the final refined model. Fig. 2 ▶ shows the quality of the corresponding maps. Whilst it is clear that the combined phases were not magically ‘correct’ (and they certainly did not allow automatic model building), they were sufficiently improved that manual rebuilding could begin. Indeed, we followed a iterative phasing protocol, each cycle of which comprised (i) building of a small additional portion of the model, (ii) refinement in *BUSTER-TNT* using the solvent-flattened combined phases from the previous cycle to define the missing-atoms envelope during refinement and (iii) calculation of a new set of combined phases within *SHARP* to help refinement of the heavy-atom model and guide the solvent flattening of maps for the next round of model building. This process eventually allowed the construction of the complete model for the fH SCR domains 6, 7 and 8 and location of the bound SOS (Prosser, Johnson, Roversi, Herbert *et al.*, 2007 ▶).

### 2.2. Case study 2: the crystal structure of a complex between SCR domains 6 and 7 from complement regulator factor H and the factor H-binding neisserial protein fHbp

The next example will also show the power of phases from a highly incomplete model to locate and aid refinement of heavy-atom sites. This example also involves a fragment of the crystal structure discussed in the previous section: the sixth and seventh SCR domains of fH were crystallized in complex with a *Neisseria meningitidis* protein whose biological function is to scavenge fH onto the bacterial surface (Schneider *et al.*, 2009 ▶). The neisserial protein fHbp is approximately twice the size of the two-domain construct fH67 and consists of a C-­terminal half (the NMR structure of which was available; Cantini *et al.*, 2006 ▶) and an N-terminal half of unknown structure. As such, this project seemed another obvious case where molecular replacement was likely to be successful: one third of the structure was known from a crystal structure (Prosser, Johnson, Roversi, Herbert *et al.*, 2007 ▶), one third was known from an NMR solution structure (Cantini *et al.*, 2006 ▶) and the final third was unknown. The crystals belonged to space group *C*2 and the unit-cell volume (Matthews, 1968 ▶) and the self-rotation function suggested that there were likely to be three copies of the complex per asymmetric unit. Initially, three copies of the fH domains could be located using each of the abovementioned *CCP*4 molecular-replacement programs; however, despite extensive searching with the NMR ensemble and models, the known portion of the fHbp structure could not be found. Even with missing-atom modelling switched on in *BUSTER-TNT* and with threefold averaging applied, it was not surprising that refinement of the fH domains alone did not yield an interpretable map for the missing fHbp components, as the partial structure only accounted for one third of the asymmetric unit.

**Figure 3:**
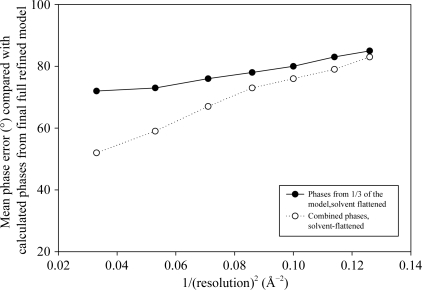
fH67–fHbp. Mean phase errors across the resolution range of the data for the *BUSTER-TNT* phases derived from the one-third model (fH67 alone) and for these phases combined with the heavy-atom phases compared with calculated phases derived from the fully refined final model.

Pt- and Hg-soaked crystals yielded diffraction data sets to a resolution of 3.2 Å; a SAD experiment collected at a wavelength of 1.8 Å gave a 3 Å resolution 28-fold redundant data set to exploit the anomalous signal from the 33 S atoms in the asymmetric unit. None of the data/signals were of sufficient quality to allow heavy-atom finding *de novo*, but using the partial model *BUSTER-TNT* phases in *SHARP* allowed the detection of six Pt sites and one Hg site, and also confirmed (with peaks higher than 3σ in *SHARP* anomalous LLG residual maps) the location of 11 of the 12 disulfides present in the fH portion of the asymmetric unit, providing a good independent corroboration of the molecular-replacement solution. *SOLOMON* solvent flattening and *DM* threefold averaging produced a map in which the majority of the fHbp C-terminal domain could be traced. Once this model had been refined (the model now being about two-thirds com­plete), additional S sites added and the phases again com­bined, the rest of fHbp could be traced: it turned out that the N-terminal domain was also a β-barrel, although differing in topology from the C-terminal one. At the end, 31 of the 33 sulfur sites were visible in the anomalous LLG maps of the long-wavelength SAD data set. Fig. 3 ▶ shows the mean phase errors of the phases derived from the third of the model before and after combination with the heavy-atom phases from the sites located using the same partial model. *Post factum* com­parison of the final structure of fHbp with the earlier NMR structure of the C-terminal barrel suggests that molecular replacement failed owing to small but significant distortions of the β-strands: the strands in the NMR structure could not all be aligned along the lengths of the crystal structure strands, so that any placement that aligned any one strand misplaced the others.

## 3. Conclusions

Using phases generated from very partial molecular-replacement models (if generated carefully) can reliably locate heavy-atom sites using either isomorphous or anomalous difference signals (even if the signal is not sufficiently strong to allow robust finding of the sites using Patterson interpretation or direct methods), with the additional advantage that using molecular-replacement phases to define the heavy-atom sub­structure avoids the need for subsequent hand determination and/or origin-choice reconciliation. Anomalous signal from disulfides can also be used as an independent check that a model placement is correct in situations where refinement statistics are ambiguous, as is generally the case with very partial models. By continuously recombining phases derived from refinement of the rebuilt structure with the experimental phasing information, potential problems of bias may be alleviated, so that rapid progress towards the final structure can be made. We have often found that trivial additions to an incomplete structure (*e.g.* of the order of ten residues added when 200 or more are still missing) can improve the phase estimates and hence the maps well enough to allow further rebuilding. Such an incremental approach can turn an apparently hopeless situation into one in which structures can be completed.

However, there is one small *caveat*: the approaches detailed here seemed at the time to be the most efficient way to complete structure determination. However, a balance should always be struck between these relatively time-intensive approaches and the potential pain/gain of going back to the laboratory to try and obtain different crystals or novel phasing information of some sort. Nevertheless, as a community, we should not forget that until recently all structures were built one residue at a time by hand and often in poor maps, and we can still do it (if we have to)!
